# A great enigma of the Italian Renaissance: paleopathological study on the death of Giovanni dalle Bande Nere (1498–1526) and historical relevance of a leg amputation

**DOI:** 10.1186/1471-2474-15-301

**Published:** 2014-09-10

**Authors:** Gino Fornaciari, Pietro Bartolozzi, Carlo Bartolozzi, Barbara Rossi, Ilario Menchi, Andrea Piccioli

**Affiliations:** Division of Paleopathology, Department of Translational Research on New Technologies in Medicine and Surgery, University of Pisa, Via Roma 57, 56126 Pisa, Italy; Past President S.I.O.T., Department of Surgery, Orthopaedic and Traumatology Clinic, G.B. Rossi Hospital, University of Verona, Piazzale Scuro 10, 37134 Verona, Italy; Department of Diagnostic and Interventional Radiology, University of Pisa, Via Paradisa 2, 56100 Pisa, Italy; Unit of Oncological Orthopaedics, Musculoskeletal Tissue Bank, Regina Elena National Cancer Institute, via Elio Chianesi 53, 00144 Rome, Italy; Department of Radiology, University of Florence, Viale Morgagni 85, 50134 Florence, Italy; Orthopedics Oncology, “Palazzo Baleani”, Teaching Hospital Policlinico Umberto I, Corso Vittorio Emanuele II 244, 00186 Rome, Italy

**Keywords:** Giovanni dalle Bande Nere, Leg amputation, Paleopathology

## Abstract

**Background:**

The Medici project consisted in archeological and paleopathological researches on some members of the great dynasty of the Italian Renaissance. The remains of Giovanni de’ Medici, so-called “dalle Bande Nere” (Forlì 1498- Mantua 1526) have not been investigated yet. The enigma of the fatal injury and leg amputation of the famous Captain excited curiosity of paleopathologists, medical scientists and Italian Society of Orthopedic and Traumatology which contributed to realize the project of exhumation and study of his skeletal remains. The aim of the study is to report the first anthropological and paleopathological results.

**Case presentation:**

The tomb of Giovanni and his wife Maria Salviati was explored and the skeletal remains were investigated. Anthropological and paleopathological examination defined: age at death, physical constitution and activity, skeletal diseases. The bones of the leg were studied macroscopically, under stereoscopic microscope, at X-ray and CT scans to detect type of injury and level of amputation.

**Conclusions:**

The skeleton and muscular insertions of Giovanni revealed a young-adult and vigorous man, subjected to stresses of military activity since adolescence. Right tibia was amputated below the proximal half of diaphysis leaving long tibio-fibular stumps with a horizontal cut only at the lateral portion. Thus, the surgeon limited to complete the traumatic hemi-amputation. Amputation in the Sixteenth Century technically consisted in guillotine incisions below the knee using crescent shaped knife and bony saw, usually leaving a quite long tibial stump. Amputations in the Sixteenth Century were contaminated and grossly performed not providing vascular binding nor wound closure. The surgeon performed the procedure in conformity with surgical knowledge of that period.

**Electronic supplementary material:**

The online version of this article (doi:10.1186/1471-2474-15-301) contains supplementary material, which is available to authorized users.

## Background

**Figure 1:**
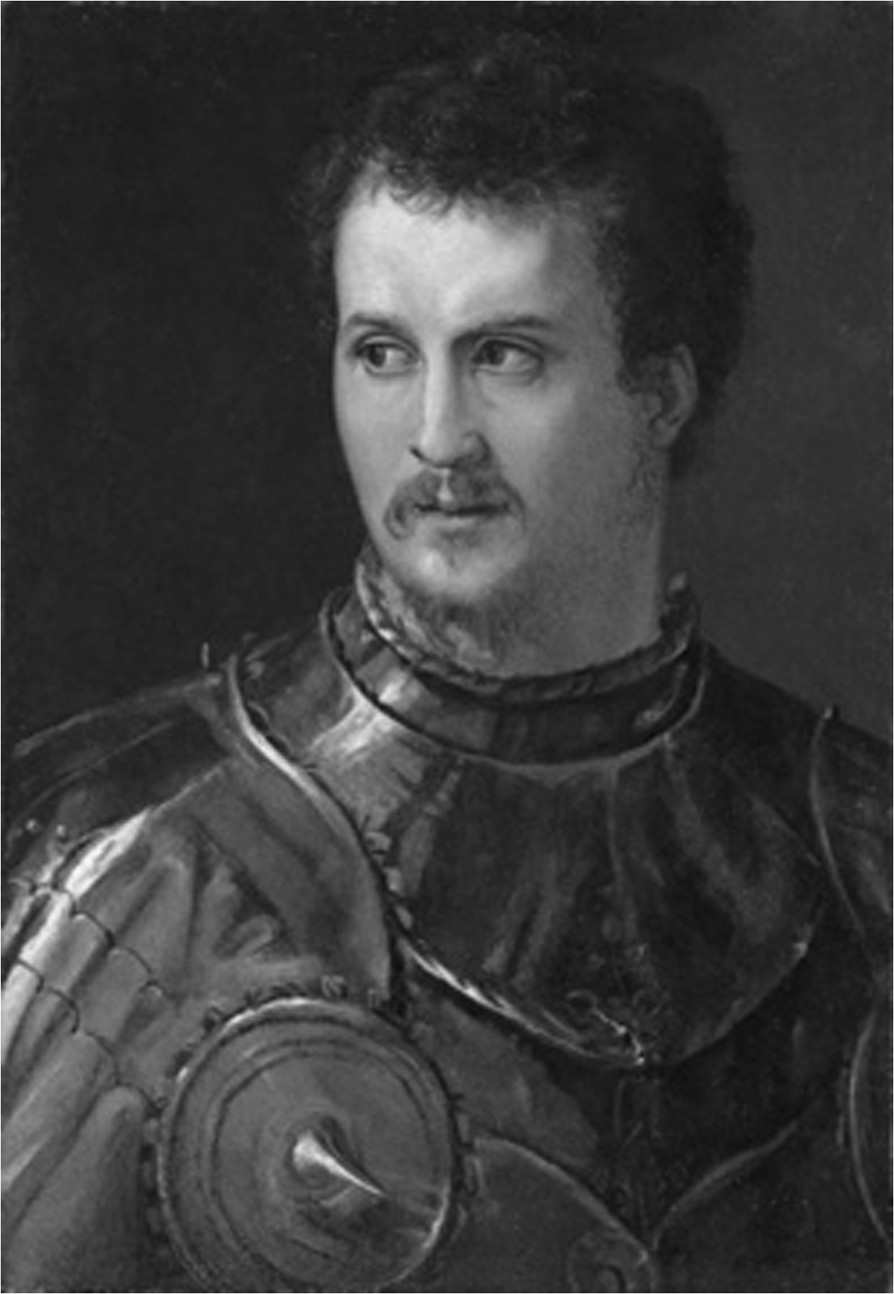
**Portrait of Giovanni dalle Bande Nere (1498–1526) (Salviati, Galleria Palatina).**

The Medici Project (2004–2007) consisted in the exhumation, exploration and paleopathological investigations on 49 tombs of the Medici family members (16th-18th centuries) housed in the church of San Lorenzo in Florence. It resulted in interesting archeological, anthropological and medical findings on the notorious family that ruled the Italian Renaissance all over three centuries [1–3]. Paleopathology is the science of morphological and molecular “footprints” left by any disease on skeletal or mummified human remains. It involves several disciplines such as history, archeology, physical anthropology, anatomopathology; when sided by archival and iconographic sources, paleopathology and history of medicine converge to research on medical therapies, epidemiological scenarios and lifestyles of ancient populations [4–6].Within the framework of the Medici Project, the enigma of the fatal injury and leg amputation of the Captain Giovanni de’ Medici, so-called “dalle Bande Nere” (Forlì 1498- Mantova 1526) (Figure 1) recently excited curiosity of paleopathologists, medical scientists and orthopedics who contributed to the realization of exhumation and study of the his remains. The “Giovanni dalle Bande Nere” project was the result of a scientific collaboration among the Division of Paleopathology of the University of Pisa, Divisions of Radiology of the Universities of Florence and Pisa and the Italian Society of Orthopaedics and Traumatology.

At the time of military Franco-Spanish conflicts, when political power was shared out by the Pope, the King of France, the Emperor of Spain and several Italian States, Giovanni dalle Bande Nere was a brave leader and a charismatic commander, beloved by his troops and feared by his enemies. Giovanni had the command of the papal troops sided with Francesco I against the Emperor Carlo V when the lansquenets, German mercenaries under Carlo V’s pay, were moving against Rome on autumn 1526: in the attempt to arrest their advance, Giovanni was injured by a ball of falconet at his right leg on November 25, 1526 near Governolo sul Po [7, 8]. After several hours from the injury, the captain was transported to Mantua to be cured by the Jew surgeon Abraham formally under the protection of marquis Federico Gonzaga, who had actually assisted the lansquenets’ descent. The wound was judged serious and infected; moreover, Giovanni had been seriously wounded at the same leg one year before by an harquebus, an episode that had implied many cures and a long period of rest. Giovanni forcedly underwent his right leg amputation and died few days later, on November 30, at 28 years of age. Several records report these events, leaving dynamics and weapon responsible for the injury, level of amputation and medical causes of death unresolved [7–12]. Discordant rumors said that Giovanni might be the victim of a political plot, thus the cut was poorly practiced by the surgeon leaving the leg stump in gangrene. The open wound was treated with plasters in use in that time, which certainly favored suppuration. The hypothesis of malarial fever or poisoning has been even reported but probably it was too late to prevent septicaemia that had probably already spread at the moment of amputation [11]. Giovanni’s corpse was first exhumed in 1857, then in 1946–47 when the armor was recovered [13–15]. The aim of the study is to report the first anthropological and paleopathological results from recent investigations on his skeletal remains.

## Case presentation

The manuscript was performed with the approval of the ethics committee of “Ministero per i beni e le attività culturali”, references number 1736–3416.07.

Skeletal remains of Giovanni dalle Bande Nere and his wife Maria Salviati were examined on November 2012. The tomb was sited in the center of the Medici Chapels in the church of San Lorenzo at Florence: floor slab was removed to reach the subterranean chamber in which the zinc coffins containing the funerary depositions of the two spouses was deposed. After an archeological survey, the box were shifted to the Lorenese Chapel, on the back of the Medici Chapel, where a temporary laboratory was organized to perform the anthropological and paleopathological examination.

**Figure 2:**
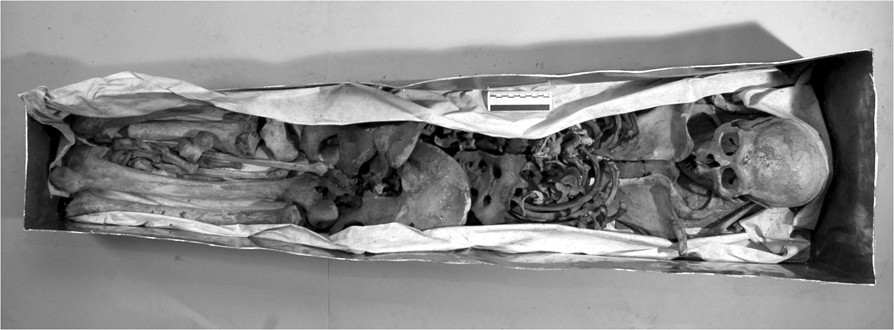
**Open funerary zinc coffin containing skeletal remains in perfect state of conservation.**

**Figure 3:**
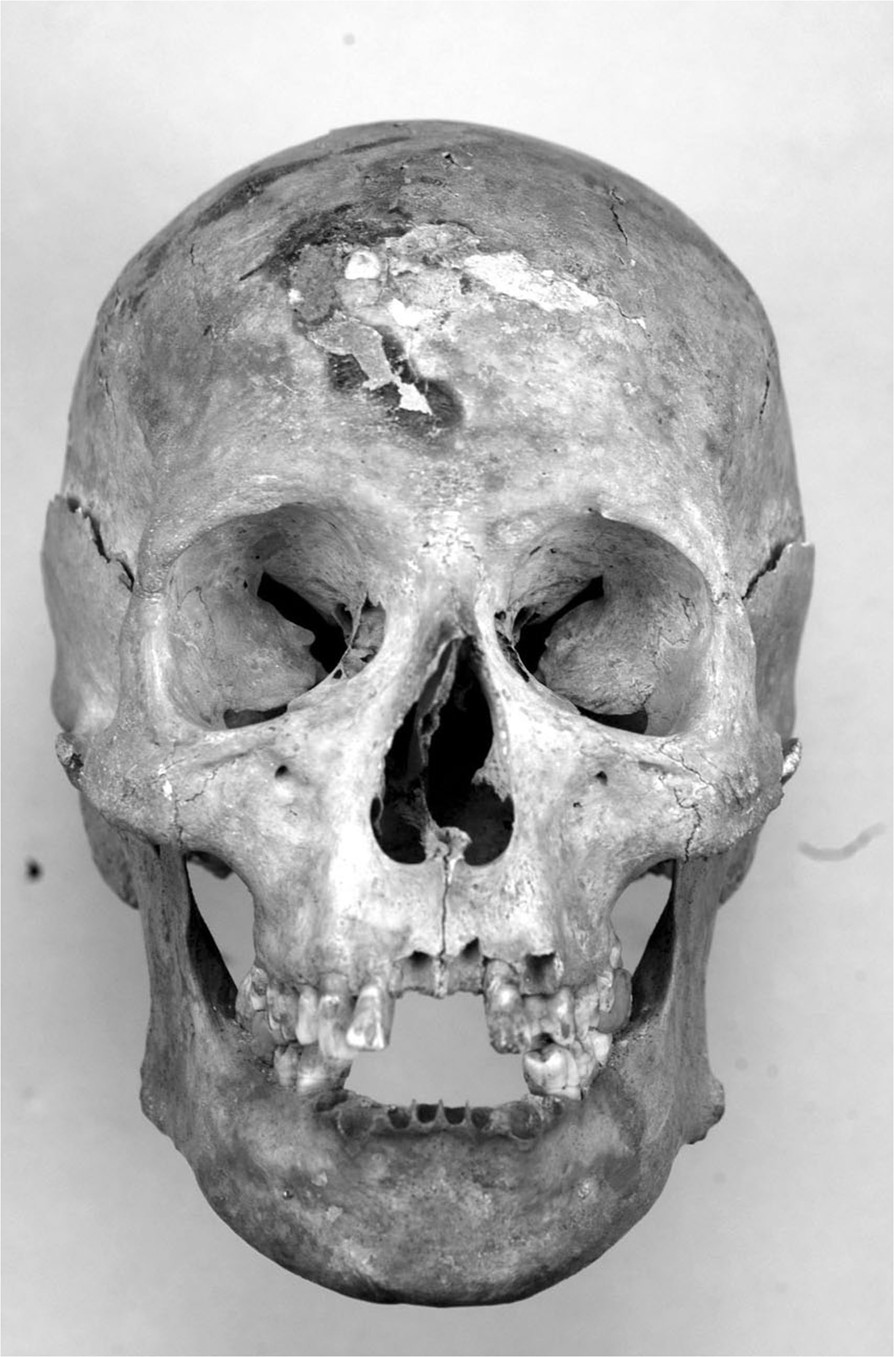
**Frontal vision of the skull of Giovanni de’ Medici.** Traumatic curvature of nasal septum is visible.

**Figure 4:**
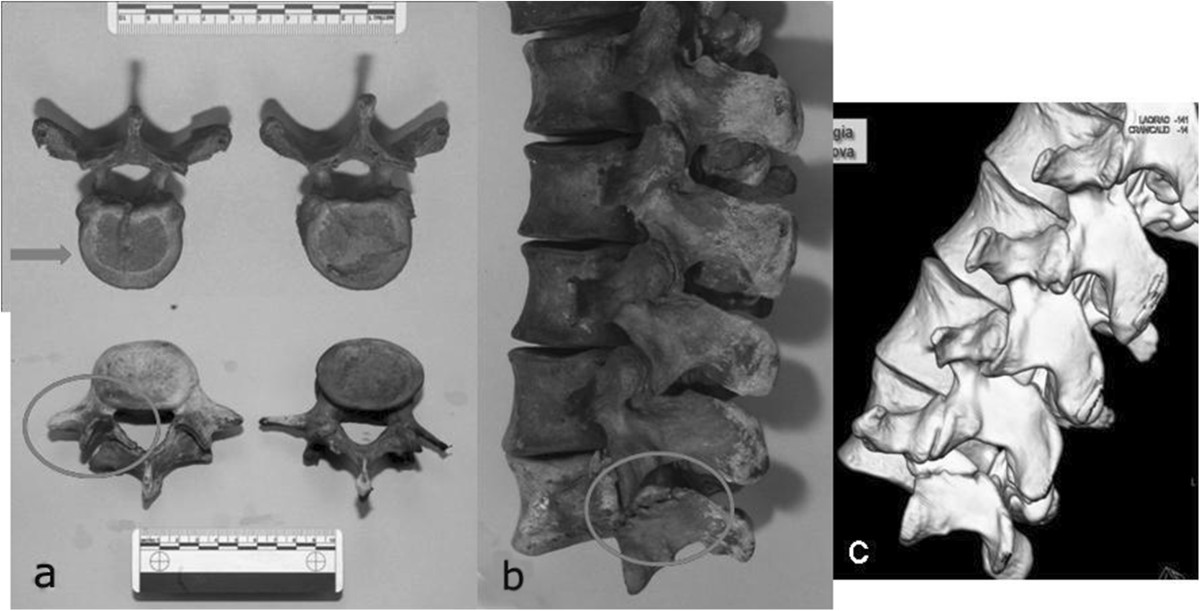
**Typical low back disorders were caused in a young horseman by cronic mechanical stresses: a Schmorl’s hearnias and b spondylolysis of the fifth lumbar vertebra, c as well-documented on CT scan.**

Anthropological investigation on Giovanni’s remains included sex identification, age at death, stature and physical constitution, examination of skeletal markers and insertions of the major muscle groups. Then, a paleopathological analysis was made in order to define some specific skeletal diseases. The bones of the amputated limb were finally examined to detect the type of injury and the exact level of amputation. Besides macroscopic observation, the skeletons were examined under a stereoscopic microscope and at X-ray and CT scans in the Department of Radiology of the Hospital of Santa Maria Nuova in Florence. The remains of Giovanni and Maria were finally reassembled and relocated in the crypt.Skeletal remains of Giovanni dalle Bande Nere appeared in good condition (Figure 2). The study of the skeleton revealed that he was a vigorous man, 1.78 m tall, with an athletic body, estimated skeletal age of 25–30 years, medium-sized skull, narrow nose and great skull capacity (1494 cc) (Figure 3). His well-developed upper limbs muscular insertions (deltoid, great pectoral, great dorsal, biceps, forearm muscles) and thigh muscles confirmed his great physical strength and robusticity. Strong hypertrophy of rotator cuff, great dorsal, teres minor and anconeus insertions were all present, as well as gluteal insertions to the femur, confirming he was a highly skilled horseman. The presence of numerous Schmorl’s hernias (Figure 4a) and a wedge collapse with spondylolysis of the fifth lumbar vertebra (Figure 4b, c) revealed that Giovanni had carried heavy loads since adolescence due to horse-riding and body armor.

**Figure 5:**
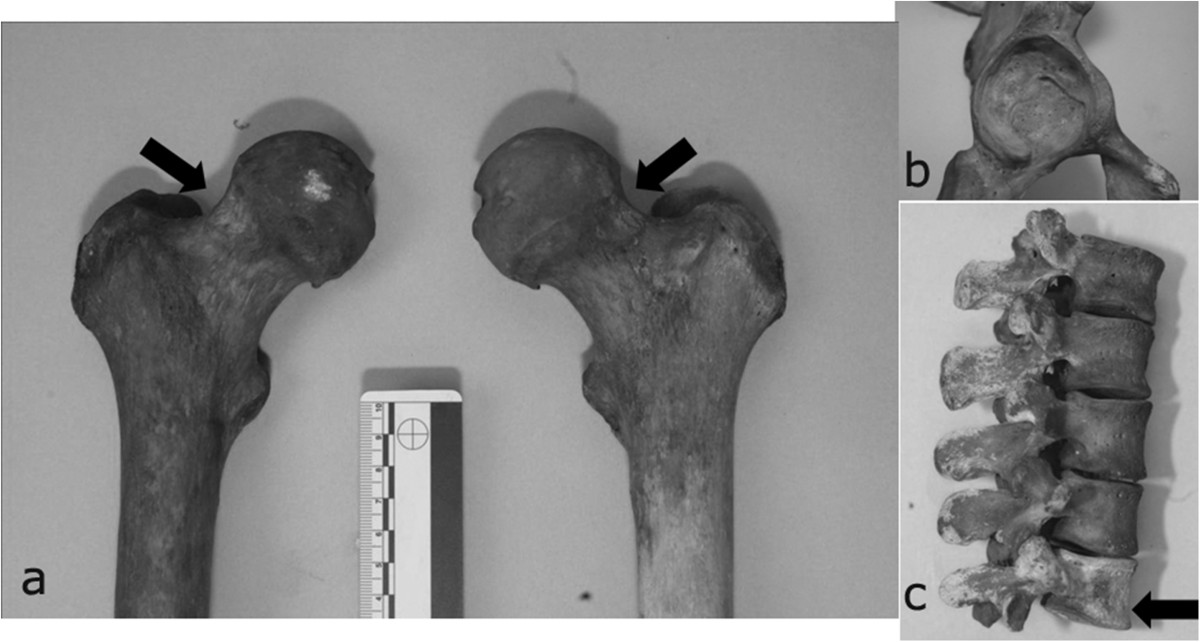
**Skeletal markers associated with habitual horseback riding are: a. Poirier’s facet, b. ovalisation of acetabula and c. wedge deformity of the body of fifth lumbar vertebra.**

**Figure 6:**
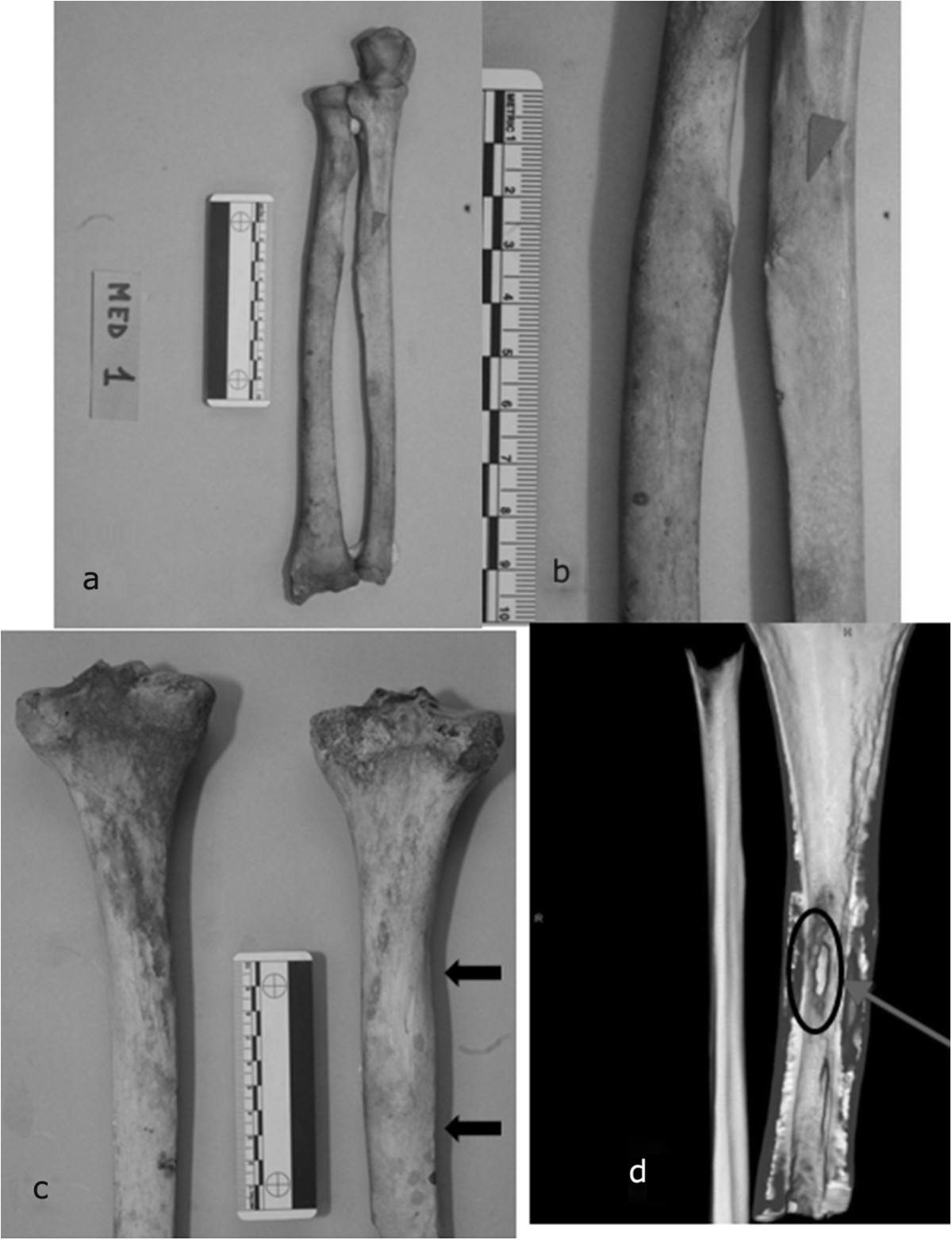
**Giovanni was injured several times in battle.** **a** Entire right radius and ulna injury. **b** Particular of the bones presenting grazes from bayonet shot. **c** Posterior surface of the right tibia with a swelling due to underlying osteomyelitic focus in reparative phase. **d** CT scan study of the tibial lesion.

Diffuse bilateral enthesitis was found at the clavicular insertions of deltoid and pectoralis major, as well as at the small trochanter (psoas muscle). Skeletal markers left by habitual horseback riding were all present: exostoses and ovalization of acetabula, hypertrophy of femoral rectum muscle, strong hypertrophy of the femoral biceps, great adductor, small and great gluteus, Poirier’s facet (Figure 5a, b and c) [16]. Paleopathological investigation showed the aftermaths of several injuries: fractures of nasal septum (Figure 3) and proximal third of the left humerus, injury from dagger affecting right ulna and radius (Figure 6a, b) and swelling of the posterior surface of the right tibia 131 (Figure 6c), with underlying osteomyelitic focus in reparative phase, as well-documented on CT (Figure 6d).

**Figure 7:**
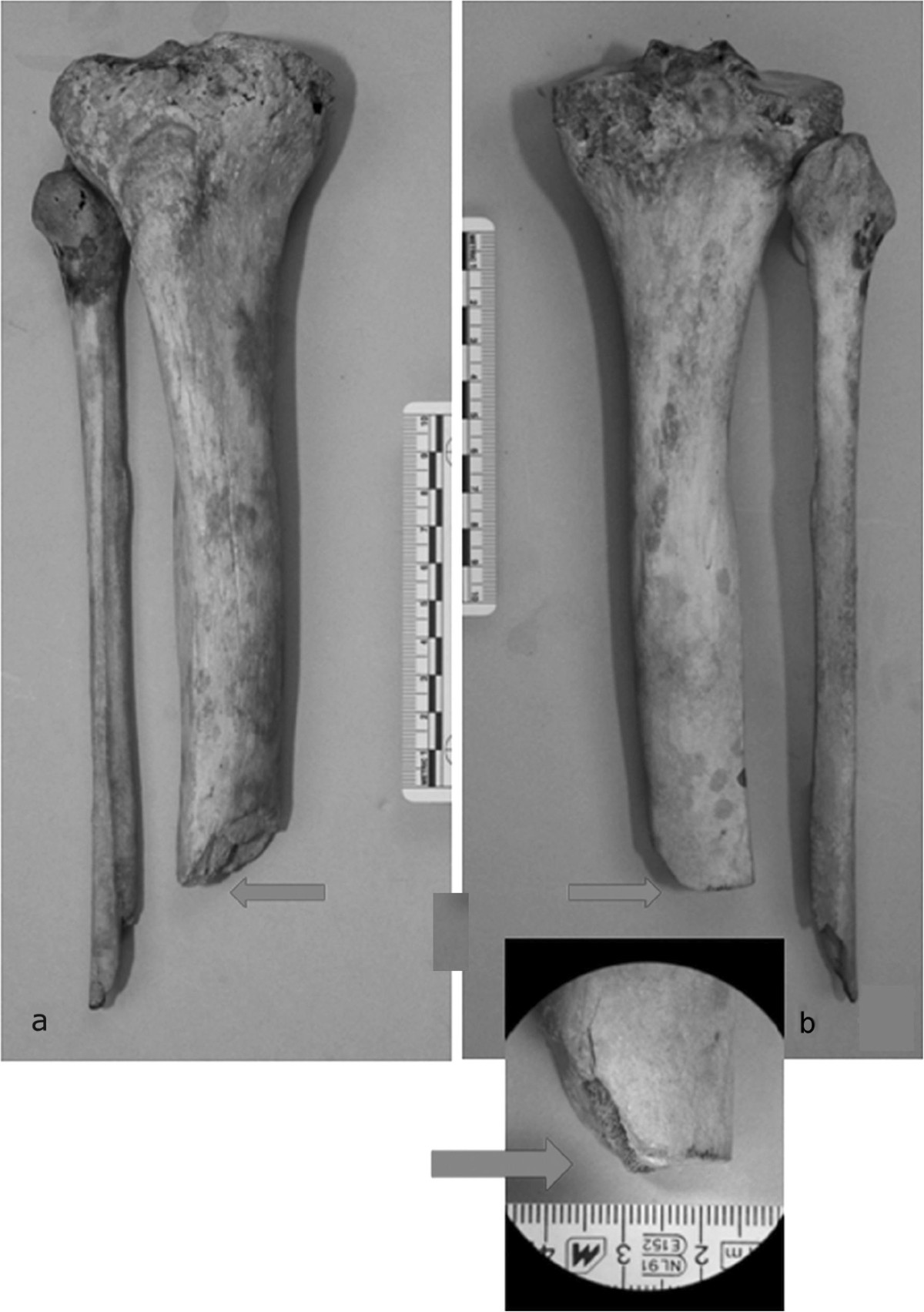
**Characteristics of amputation were investigated: a anterior and b posterior view of right tibia and fibula reveal the injury from falconet cannonball at the same level of the horizontal surgical cut (arrow).**

**Figure 8:**
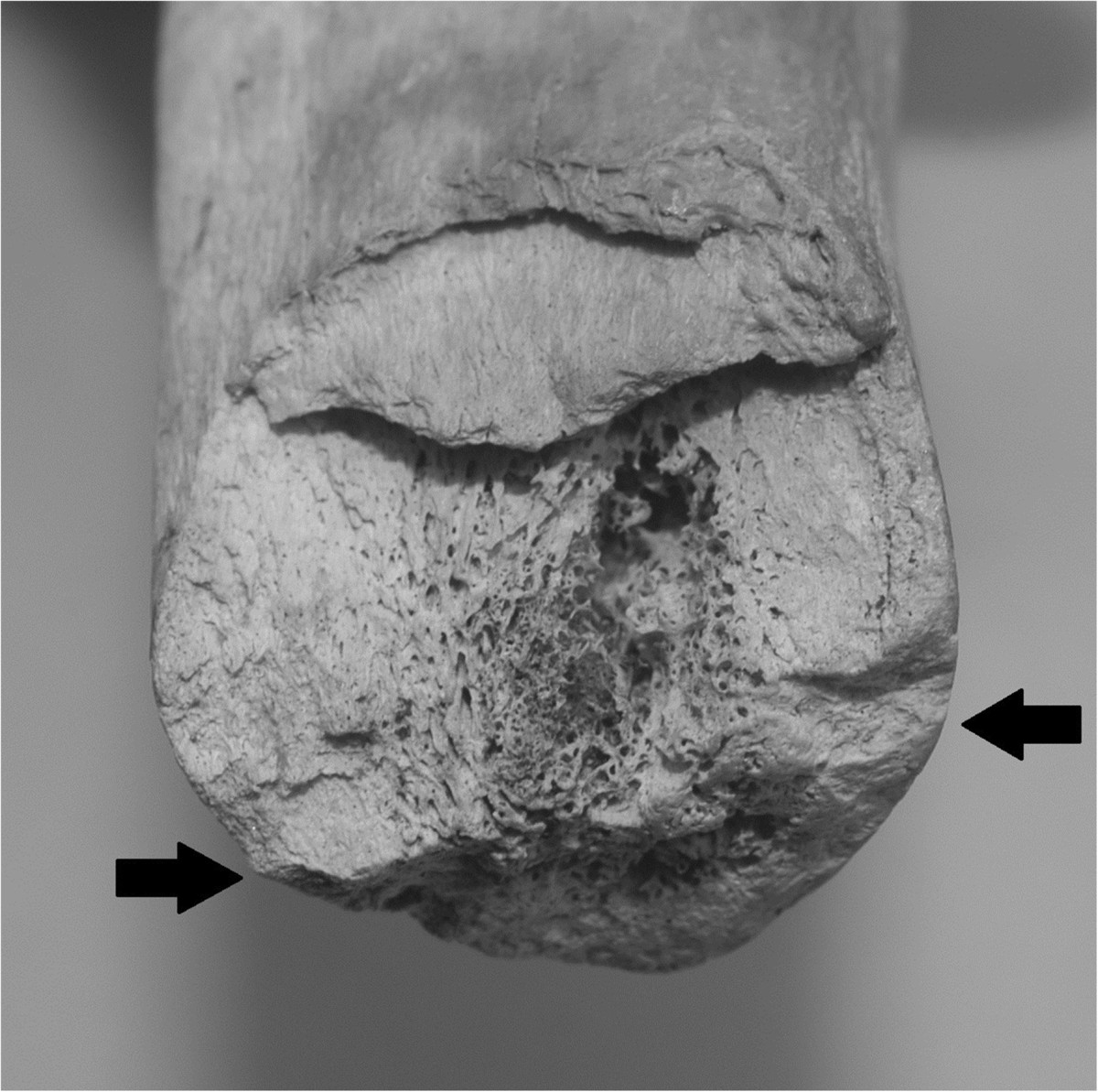
**Proximal stump of right tibia with the oblique fracture of the ball of falconet (above the arrows) and the cut of surgical amputation (below the arrows), at the stereomicroscope.**

The amputation level was exactly assessed: the tibia was sawn immediately below the proximal half of diaphysis and only the lateral portion was surgically treated with a horizontal cut (Figure 7a, b, arrow). Only oblique splitting was found at the medial site of the tibia. At stereoscopic microscope, surgical section revealed a marked proliferation of endosteal callus, due to the previous harquebus shot wound occurred about one year before the death; distal extremity of fibular fragment showed an oblique splitting and a horizontal cut, with no sign of reparative process in the medullar canal (Figure 8). Considering the morphological aspect of the tibial and fibular injury, it was probably due to a cannonball from a falconet of caliber 6–7 cm, as written by Benedetto Agnello in the same day of injuring [8]. The limb had been severely damaged by a traumatic hemi-amputation when surgeon Abramo performed the intervention, consisting in a simple completion of the amputation and regularization of proximal fragments.

## Conclusions

Giovanni dalle Bande Nere is a central figure of the Italian Renaissance. He was son of Giovanni de’ Medici and Caterina Sforza, nephew of the Popes Leone X and Clemente VII, both named de’ Medici, father of the first Gran Duke of Tuscany Cosimo I. He was at the center of the genealogic tree of the epic Florentine family and in the middle of the Franco-Spanish wars that took place in northern Italy in the early decades of the Sixteenth century. His military talent unfelt when the traditional heavy cavalry and steel weapons were abandoned for firearms, including harquebus, muskets and guns. The historical and orthopedic interests of the “Giovanni dalle bande Nere” project arise from the halo of mystery about his violent death, which dynamics and causes have been unknown for a long time.

The Medici project previously demonstrated that many components of the family were affected by several illnesses, abscesses, malarial fevers, arthritis, Diffuse Idiopathic Skeletal Hyperostosis and familiar arthropathy [17–19]. On the contrary, this recent paleopathological study on Giovanni dalle Bande Nere revealed interesting orthopedic findings and the originality of this figure among the Medici dynasty for his untimely death, when he was still young and healthy. Archival records about the weapon that caused the fatal injury are often discordant, probably because it was mistaken for the harquebus that had shut Giovanni at the same leg one year before [10, 11]; moreover, harquebus and muskets were the most commonly used firearms of that period [7, 12]. Other sources report that Giovanni was shot from a cannonball, since the Duke of Ferrara Alfonso I betrayed papal troops selling three falconets to lansquenets: the river traffic of arms was secretly favored by hiding falconets among provisions to imperial army [7, 8]. Historical evidences refer the wound at the leg, sometimes proximally to the foot, other times around the knee but surely more than 20 hours passed from the injury to first aids; therefore, the injury might have involved also the vascular bundle along with the crush fracture [7–9, 11, 12, 20].

Giovanni arrived at Mantua in critical conditions and gangrene compelled the surgeon Abraham to perform the amputation. Amputation in the Sixteenth Century technically consisted in guillotine incisions below the knee using crescent-shaped knife and bony saw, usually leaving a quite long tibial fragment; afterwards, Ambroise Paré defined the stump length in 5 fingers (10 cm) below the knee [21]. Vascular binding was not provided, whereas haemostasis was performed through the practice of cauterization; however, the cautery was itself a means of infection [21]. Paleopathological investigations lead to exclude the hypothesis of an amputation above the knee, since the surgeon Abraham performed the procedure as better as he could in conformity with surgical knowledge of that period [7, 21]. The reason for which he left tibial and fibular stumps longer than normal remains unknown: was it in consideration of a future prosthesis? During the Middle Ages and Renaissance limb prostheses were made in iron, steel, copper or wood, locked with screws or strings in fixed positions. However, lower limb prostheses could poorly allow walking and weight-bearing despite an esthetic role in order to hide deformity and mutilation while riding during the battle. Just few years after Giovanni’s death, in 1536, Ambroise Paré projected a above the knee prosthesis with joint articulation and proximal notch similar to modern prostheses [21]. Otherwise, the leg so inexorably damaged that were hemi-amputation and gangrene fatal in any case?

During the three days after amputation, Giovanni alternated between delirium and comatose phases, due to malarial fever or else to sepsis; this might have led to the hypothesis of poisoning to endorse the theory of a political plot [11]. Since the first exhumation of Giovanni dalle Bande Nere in 1857, death was attributed to the imperfection of surgical amputation, describing the tibial section as the result of a coarse procedure using “a carpenter’s saw” [13].

This paper reports only the preliminary results of the investigations on Giovanni’s skeletal remains: further laboratory studies are still in progress. Some bone samples will be taken for laboratory immunological tests, ancient DNA, immunochromatographic tests, already experimented to other Medici samples, for the diagnosis of malaria, disease attested by the historical sources in the months preceding the death of Giovanni [1, 11, 14].

This study shows that the integration between history of medicine and paleopathology can bring historically important figures to life: scientific methods of research are used to discover their disease, lifestyle habits, their personality, in other words the true story of the past.

## Consent

Written informed consent was obtained from the “Ministero per i beni e le attività culturali” for publication of this case report and any accompanying images. A copy of the written consent is available for review by the Editor of this journal.

## Authors’ original submitted files for images

Below are the links to the authors’ original submitted files for images. Authors’ original file for figure 1 Authors’ original file for figure 2 Authors’ original file for figure 3 Authors’ original file for figure 4 Authors’ original file for figure 5 Authors’ original file for figure 6 Authors’ original file for figure 7 Authors’ original file for figure 8

## References

[1] Fornaciari G, Vitiello A, Giusiani S, Giuffra V, Fornaciari A, Villari N (2007). The Medici Project first anthropological and paleopathological results of the exploration of the Medici tombs in Florence. Med Secoli.

[2] Fornaciari G (2009). Il Progetto Medici: primi risultati dello studio paleopatologico dei Granduchi di Toscana (secoli XVI-XVIII). Arch Antrop Etnol.

[3] Villari N, Fornaciari G, Lippi D, Cerinic MM, Ginestroni A, Pellicanò G, Mascalchi M (2009). Scenes from the past: the Medici Project radiographic survey. Radiographics.

[4] Aufderheide AC, Rodriguez-Martin C (1998). The Cambridge Encyclopedia of Human Paleopathology.

[5] Ortner DJ (2003). Identification of Pathological Conditions in Human Skeletal Remains.

[6] Waldron T (2008). Palaeopathology.

[7] Guicciardini F (1971). Storia d’Italia.

[8] Gobbetti C (1987). Governolo, un viaggio nella storia: le guerre, la chiesa, il fiume. Lettera di Benedetto Agnello, ambasciatore presso la Repubblica di Venezia, al Marchese di Mantova, 25 novembre 1526.

[9] Corazzini GO (1906). Ricordanze di Bartolomeo Masi calderaio fiorentino dal 1478 al 1526.

[10] Mecatti GM (1755). Storia Cronologica della città di Firenze o siano Annali della Toscana.

[11] de Rossi G (1833). Vita di Giovanni de’ Medici.

[12] Lapini A: Diario fiorentino di Agostino Lapini dal 252 al 1596. Edited by: Sansoni GC. 1900, Firenze: IT, 94-

[13] Sommi PG (1888). Esumazione e ricognizione delle ceneri dei Principi Medicei fatta nell’anno 1857. Processo verbale e note. Arch Stor Ital.

[14] Genna G (1948). Ricerche antropologiche sulla famiglia dei Medici. Atti Accad Naz Lincei.

[15] Lippi D (2006). Illacrimate sepolture. Curiosità e ricerca scientifica nella storia delle riesumazioni dei Medici.

[16] Belcastro MG, Facchini F, Neri R, Mariotti V (2001). Skeletal markers of activity in the early Middle Ages necropolis of Vicenne-Campochiaro (Molise, Italy). J Paleopath.

[17] Fornaciari G, Giuffra V, Giusiani S, Villari N, Vitiello A (2009). The “Gout” of the Medici, Grand Dukes of Florence: a Palaeopathological Study. Rheumatology.

[18] Giuffra V, Giusiani S, Fornaciari A, Villari N, Vitiello A, Fornaciari G (2010). Diffuse Idiopathic Skeletal Hyperostosis in the Medici, Grand Dukes of Florence (XVI century). Eur Spine J.

[19] Giuffra V, Vitiello A, Giusiani S, Fornaciari A, Villari N, Fornaciari G (2010). Spinal pathology in the Medici family, Grand Dukes of Florence (XVI-XVII centuries). Paleopat Newsl.

[20] Braghirolli W, d’Arco C: Documenti inediti intorno a Maestro Abramo, medico mantovano del secolo XVI. Edited by: Segna L. 1867, Mantova: IT

[21] Kirkup J (2007). A History of Limb Amputation.

[CR22] The pre-publication history for this paper can be accessed here:http://www.biomedcentral.com/1471-2474/15/301/prepub

