# Supplementary figures and images for: A great enigma of the Italian Renaissance: paleopathological study on the death of Giovanni dalle Bande Nere (1498–1526) and historical relevance of a leg amputation
Source: BMC Musculoskelet Disord. 2014 Sep 10;15:301. doi: 10.1186/1471-2474-15-301 (PMC4246521; doi:10.1186/1471-2474-15-301)

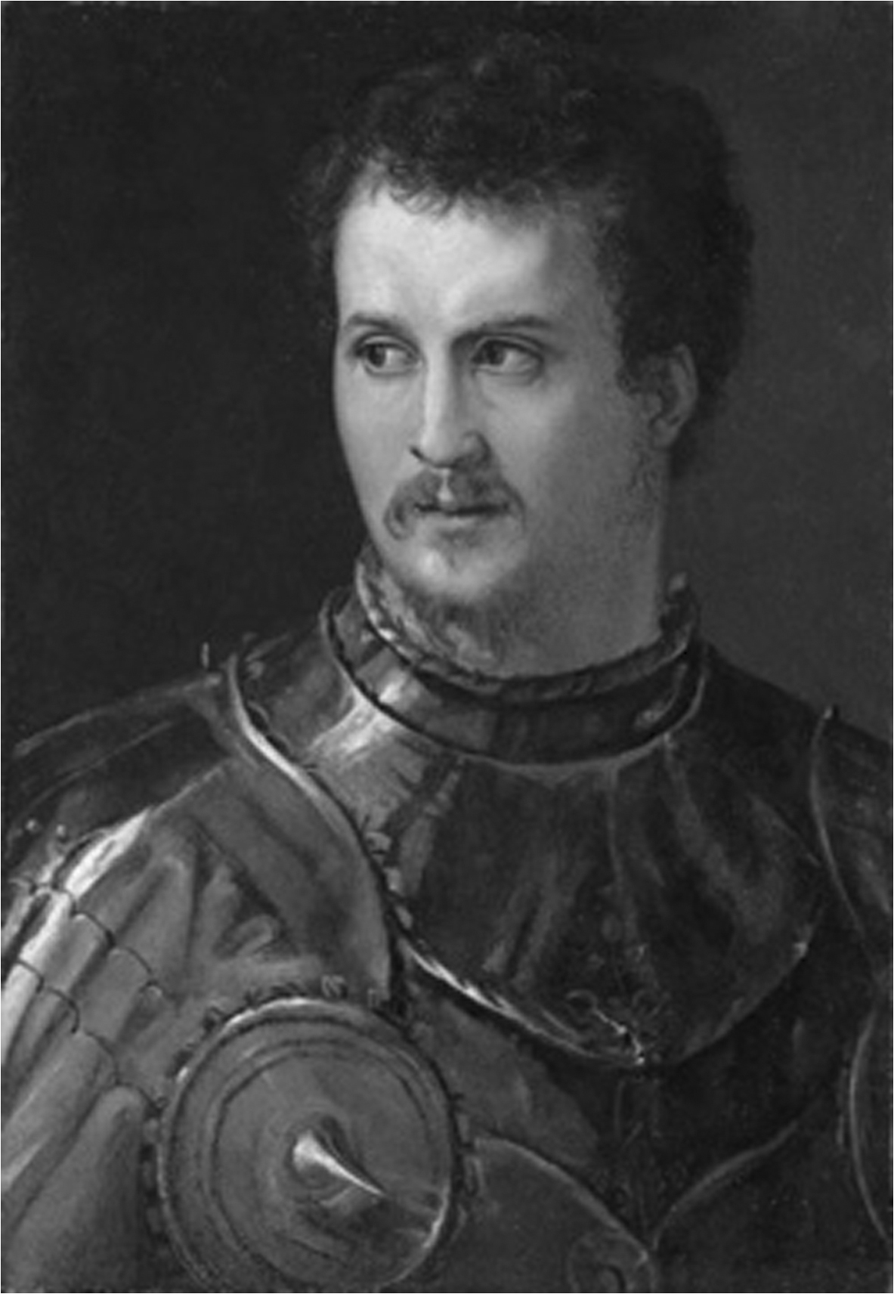

Supplement: Supplementary file 1 — Authors’ original file for figure 1 [file 12891_2014_2319_MOESM1_ESM.tif]

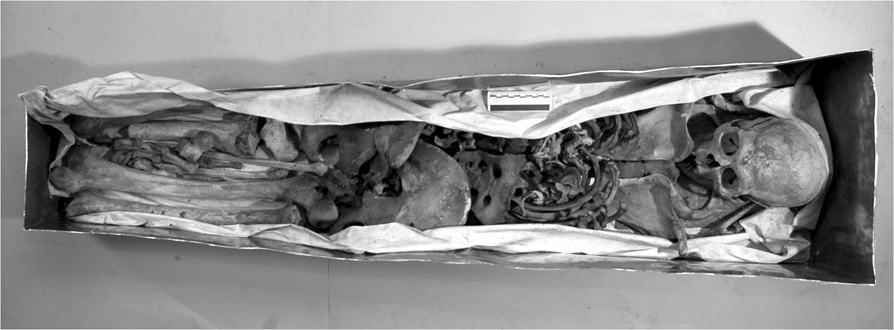

Supplement: Supplementary file 2 — Authors’ original file for figure 2 [file 12891_2014_2319_MOESM2_ESM.tif]

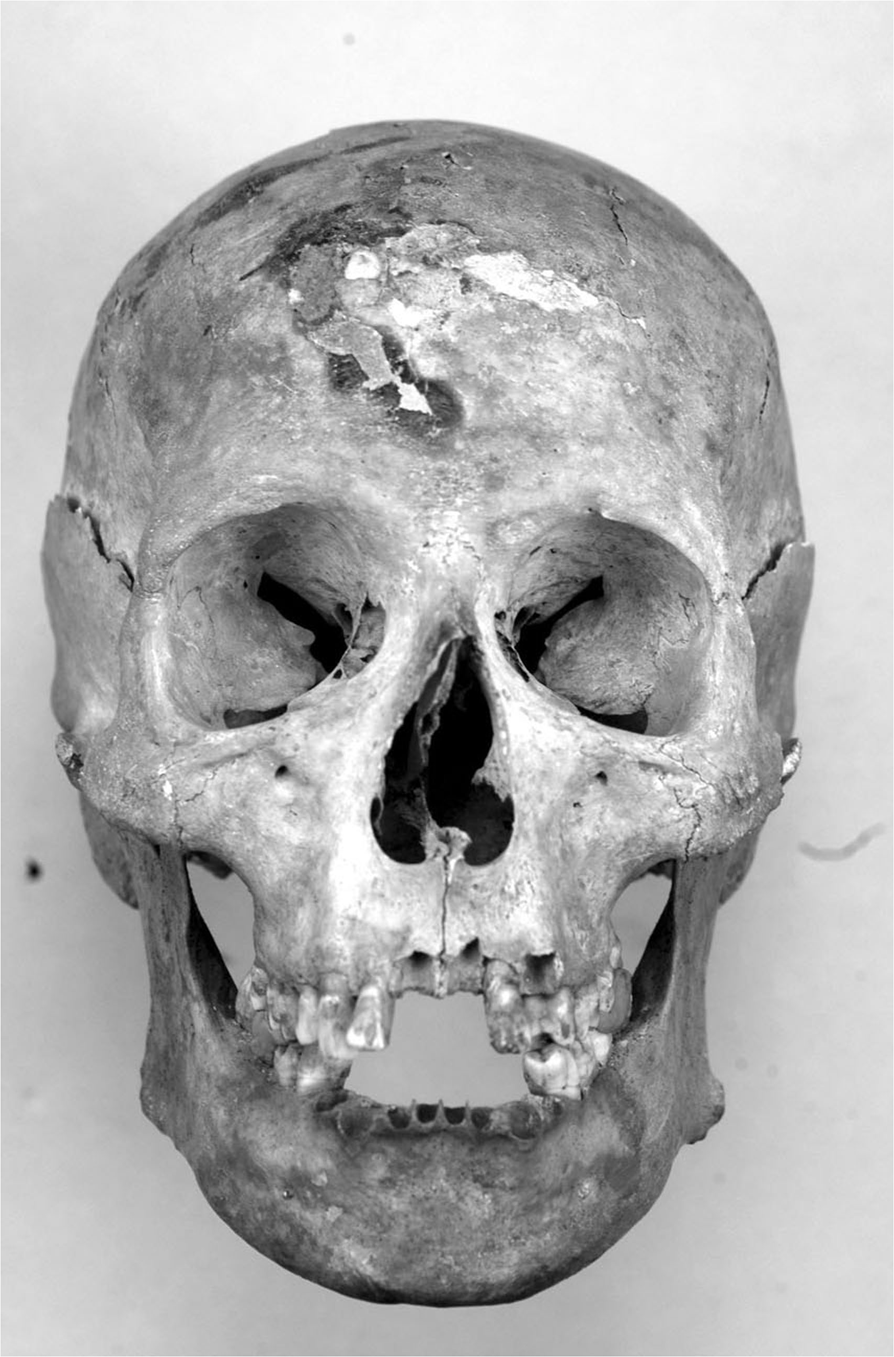

Supplement: Supplementary file 3 — Authors’ original file for figure 3 [file 12891_2014_2319_MOESM3_ESM.tif]

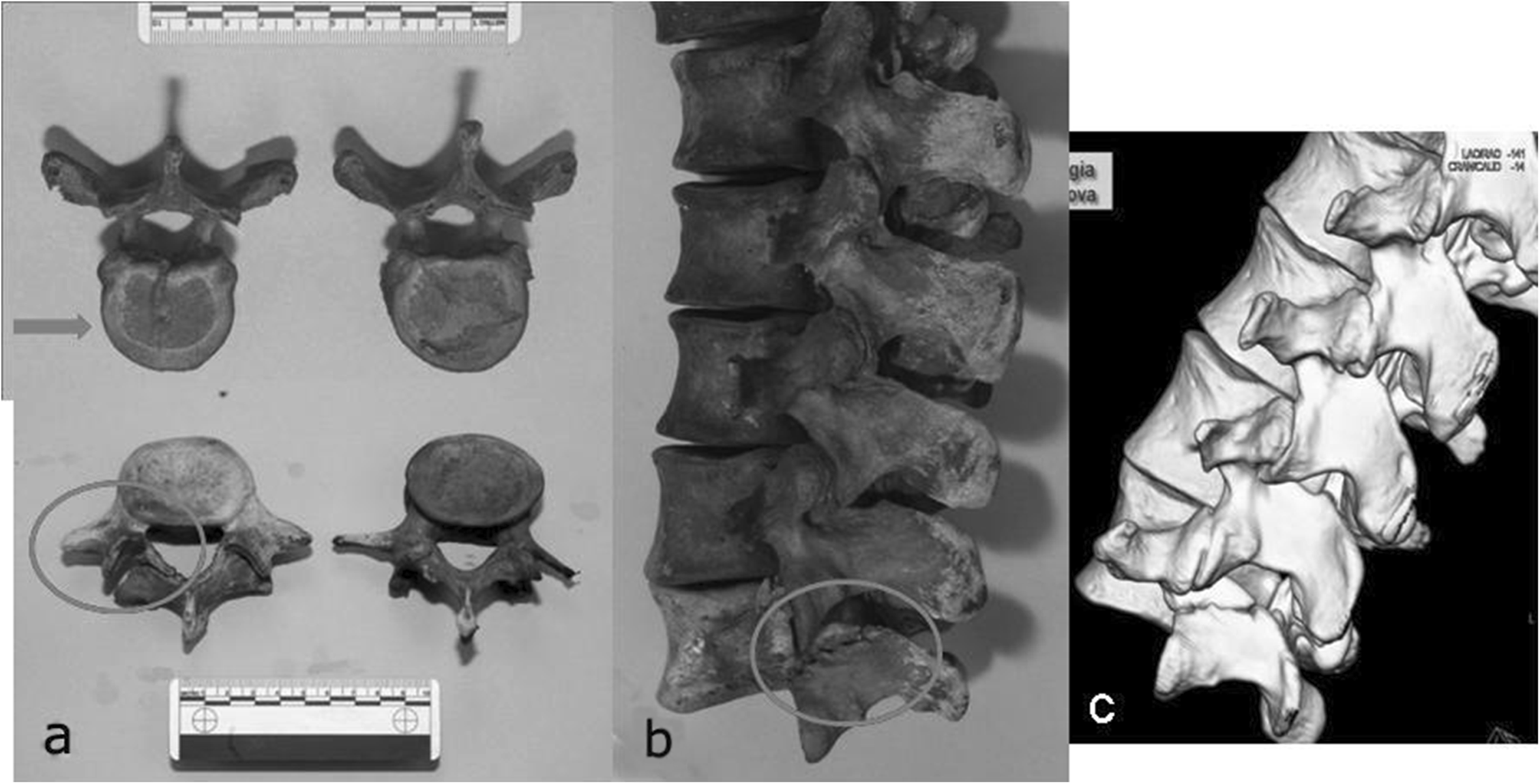

Supplement: Supplementary file 4 — Authors’ original file for figure 4 [file 12891_2014_2319_MOESM4_ESM.tif]

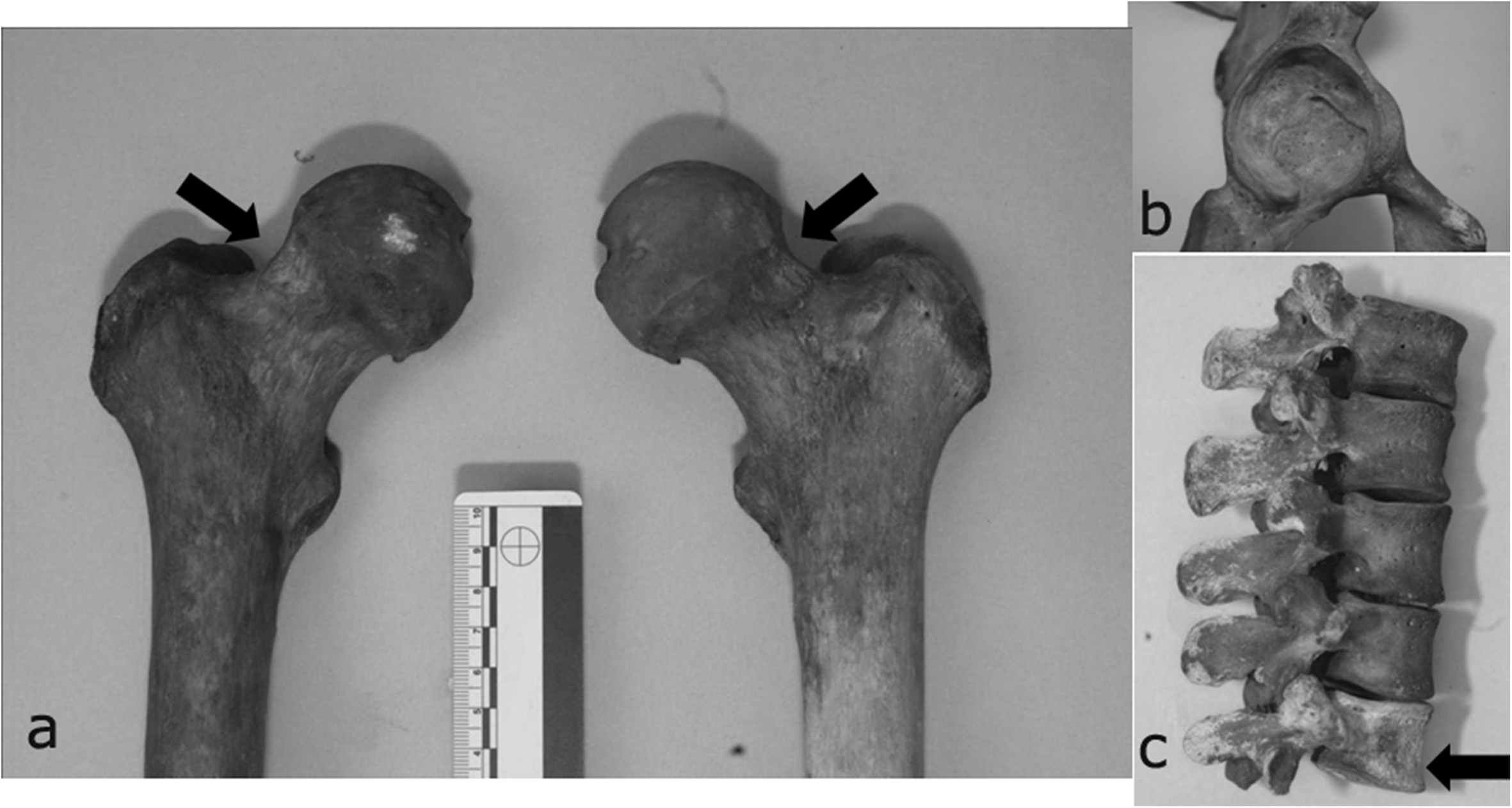

Supplement: Supplementary file 5 — Authors’ original file for figure 5 [file 12891_2014_2319_MOESM5_ESM.tif]

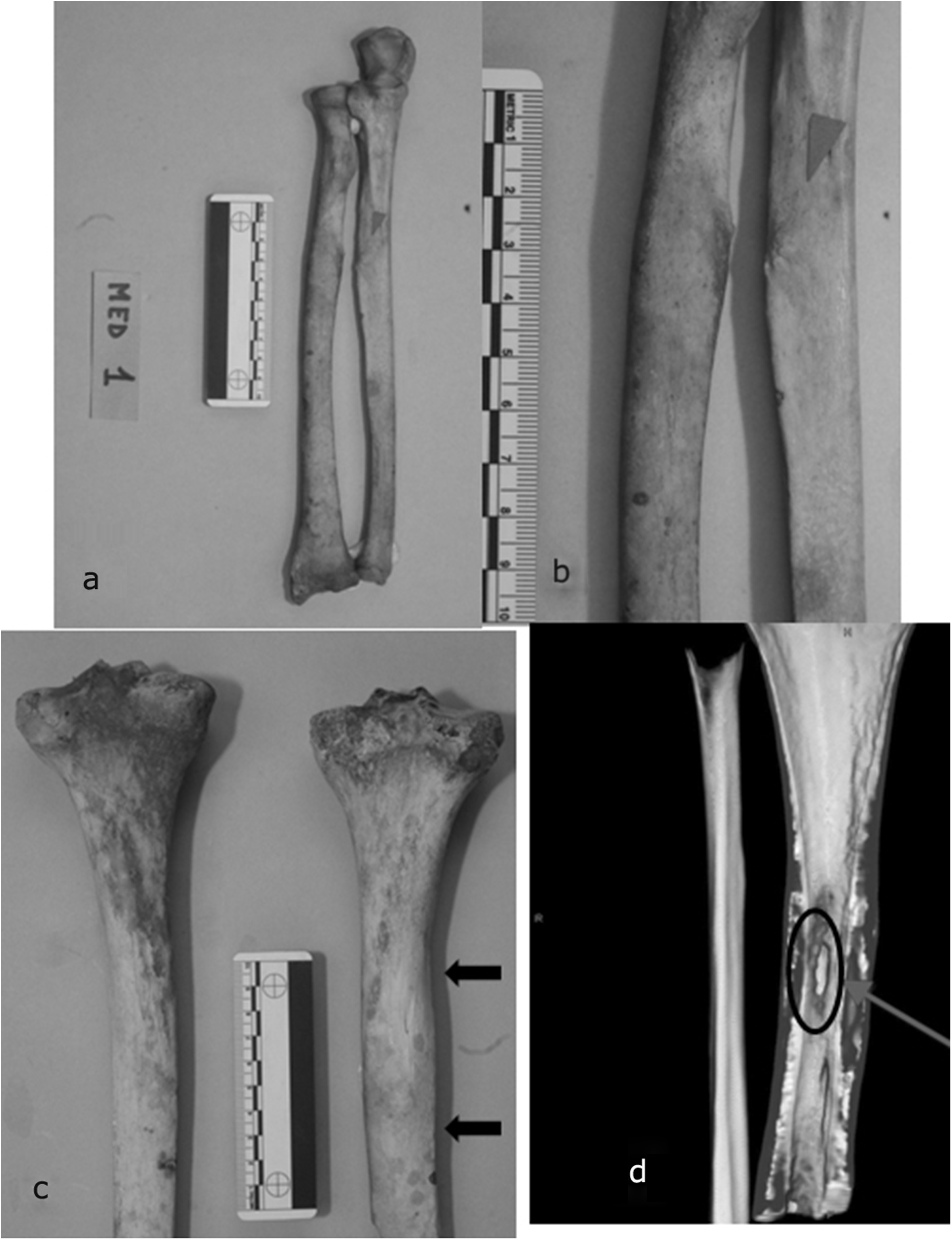

Supplement: Supplementary file 6 — Authors’ original file for figure 6 [file 12891_2014_2319_MOESM6_ESM.tif]

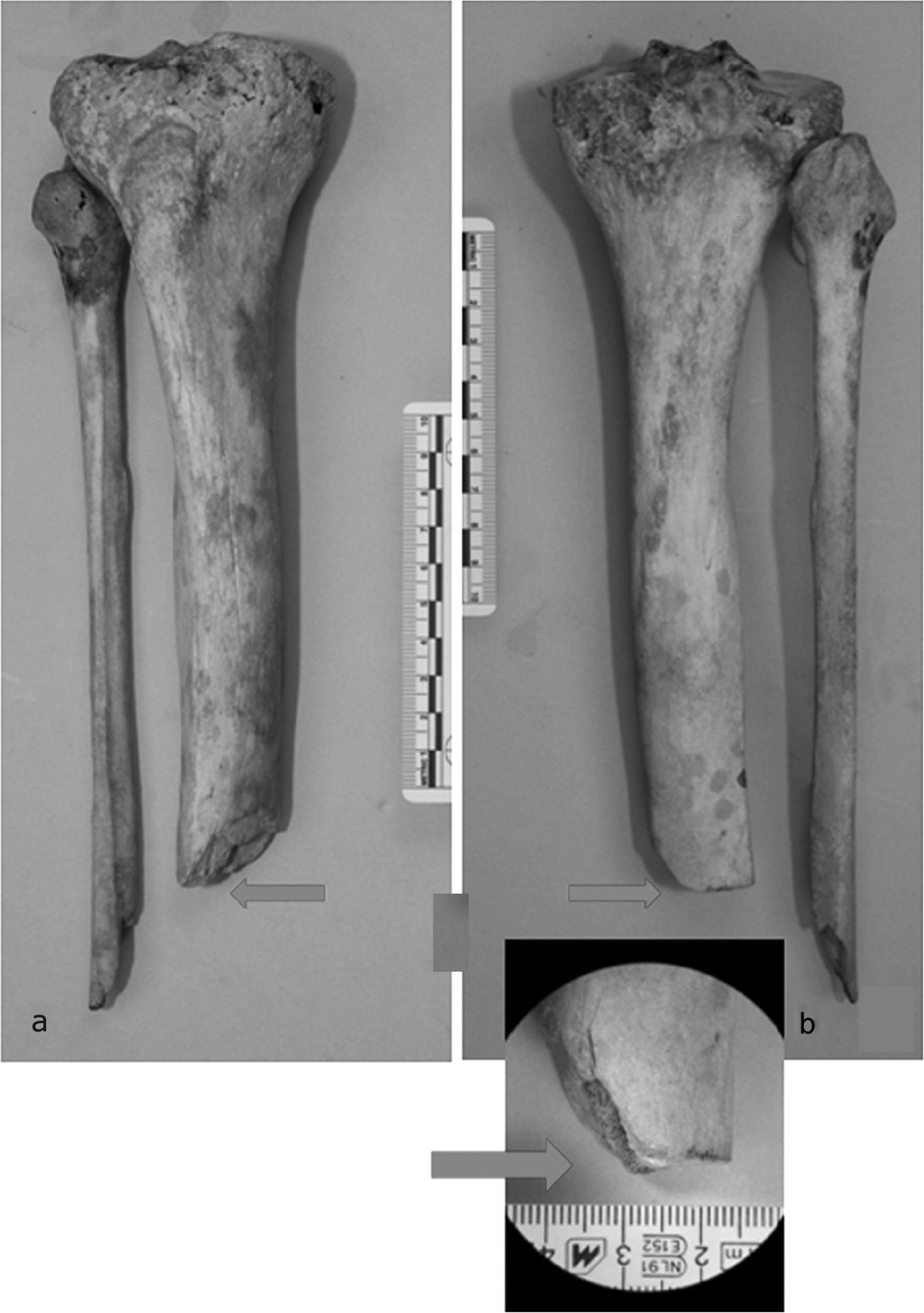

Supplement: Supplementary file 7 — Authors’ original file for figure 7 [file 12891_2014_2319_MOESM7_ESM.tif]

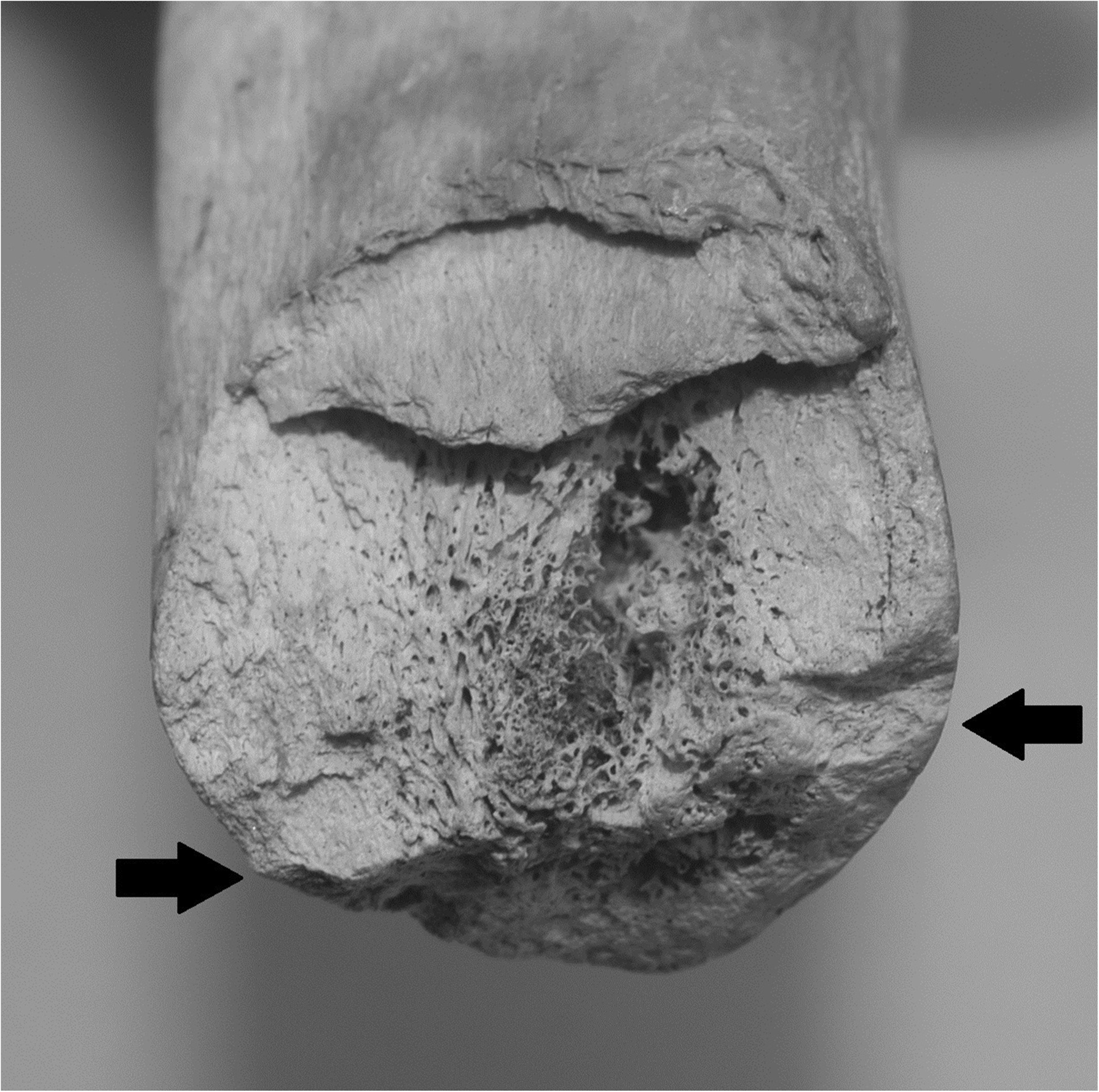

Supplement: Supplementary file 8 — Authors’ original file for figure 8 [file 12891_2014_2319_MOESM8_ESM.tif]
